# Serum KIAA1199 is an advanced-stage prognostic biomarker and metastatic oncogene in cholangiocarcinoma

**DOI:** 10.18632/aging.103964

**Published:** 2020-11-10

**Authors:** Xiangyu Zhai, Wei Wang, Yunlong Ma, Yijia Zeng, Dandan Dou, Haoning Fan, Jianping Song, Xin Yu, Danqing Xin, Gang Du, Zhengchen Jiang, Hao Zhang, Xinlu Zhang, Bin Jin

**Affiliations:** 1 Department of Surgery, School of Medicine, Cheeloo College of Medicine, Shandong University, Jinan, China; 2 Department of General Surgery, Qilu Hospital of Shandong University, Jinan, China; 3 Radiology Department, Qilu Hospital of Shandong University, Jinan, China; 4 School of Basic Medical Sciences, Shandong University, Jinan, China; 5 College of Traditional Chinese Medicine, Southern Medical University, Guangzhou, China; 6 Ji Nan Central Hospital, Jinan, China

**Keywords:** cholangiocarcinoma, KIAA1199, serum, bile, biomarker

## Abstract

Background: Cell proliferation and migration are the determinants of malignant tumor progression, and a better understanding of related genes will lead to the identification of new targets aimed at preventing the spread of cancer. Some studies have shown that KIAA1199 (CEMIP) is a transmembrane protein expressed in many types of noncancerous cells and cancer cells. However, the potential role of KIAA1199 in the progression of cholangiocarcinoma (CCA) remains unclear.

Results: Analysis of cancer-related databases showed that KIAA1199 is overexpressed in CCA. ELISA, immunohistochemistry, Western blotting and qPCR indicated high expression levels of KIAA1199 in serum, CCA tissues and CCA cell lines. In the serum (n = 41) and large sample validation (n = 177) cohorts, higher KIAA1199 expression was associated with shorter overall survival and disease-free survival times. At the cellular level, KIAA1199 overexpression (OE) promoted CCA growth and metastasis. Subcutaneous tumor xenograft experiments showed that KIAA1199 enhances CCA cell proliferation. Additionally, the expression levels of components in the EMT-related TGF-β pathway changed significantly after KIAA1199 upregulation and silencing.

Conclusion: KIAA1199 is a promising new diagnostic molecule and therapeutic target in CCA. The serum KIAA1199 level can be used as a promising clinical tool for predicting the overall postoperative outcomes of patients with CCA.

Methods: CCA-related KIAA1199 data were downloaded from the Gene Expression Omnibus (GEO) and The Cancer Genome Atlas (TCGA) databases. To assess the prognostic impact of KIAA1199, an enzyme-linked immunosorbent assay (ELISA) was used to measure the serum level of KIAA1199 in 41 patients who underwent surgical resection. Immunohistochemical staining, Western blotting and qPCR were used to verify and retrospectively review the expression levels of KIAA1199 in cancer tissue specimens from 177 CCA patients. The effect of KIAA1199 on CCA was evaluated by cell-based functional assays and subcutaneous tumor xenograft experiments. The expression levels of proteins associated with epithelial-mesenchymal transition (EMT) and activation of relevant signaling pathways were measured via Western blotting.

## INTRODUCTION

Cholangiocarcinoma (CCA) is an epithelial malignancy that occurs in different parts of the biliary tree and constitutes the second most common primary hepatic malignancy. CCA can be classified into three main types according to its anatomical location: perihepatic, distal and intrahepatic CCA (pCCA, dCCA and iCCA, respectively) [1]. The incidence of CCA is lower in Western countries (0.1-0.2/100,000 population) than in Southeast Asia (50-113/100,000 population), and CCA affects more males than females [2]. Currently, surgical lesion removal is a common curative treatment for CCA. Because of the asymptomatic nature of early-stage CCA, most patients are diagnosed at an advanced stage [3]. Surgical resection does not significantly improve long-term survival, and the rates of recurrence and metastasis are high [4–8]. Therefore, identification of a biomarker associated with CCA metastasis and prognosis is critical for the treatment of CCA. These biomarkers can serve as potential diagnostic molecules and therapeutic targets in CCA.

KIAA1199 (CEMIP, https://www.ncbi.nlm.nih.gov/gene/57214) is a protein indicator of cell migration that is localized to the perinuclear space (likely the ER) and cell membrane and is encoded by a gene located on chromosome 15q25.1 [9–11]. A related study showed that patients developed hearing loss due to mutations in KIAA1199 [12]. Moreover, severe tumor invasion and uncontrolled proliferation are associated with KIAA1199 overexpression (OE). Several studies have supported these observations in various cancers, including gastric carcinoma [13], breast carcinoma [14], colorectal tumors [15], hepatocellular carcinoma [16], prostate carcinoma [17] and oral squamous cell carcinoma [18]. In addition, these studies identified the clinical relevance of KIAA1199 to cancer (e.g., disease stage/5-year survival rate), but in most of the studies, the cohort was too small to allow any meaningful conclusions to be drawn. Moreover, KIAA1199 has been found in cell supernatants [19]. Therefore, KIAA1199 may be a secretory factor that promotes tumor development. In this study, we examined the expression level of KIAA1199 in CCA and sought to determine whether serum KIAA1199 levels can be used as a postoperative prognostic indicator.

## RESULTS

### Public database forecast results

**Figure 1:**
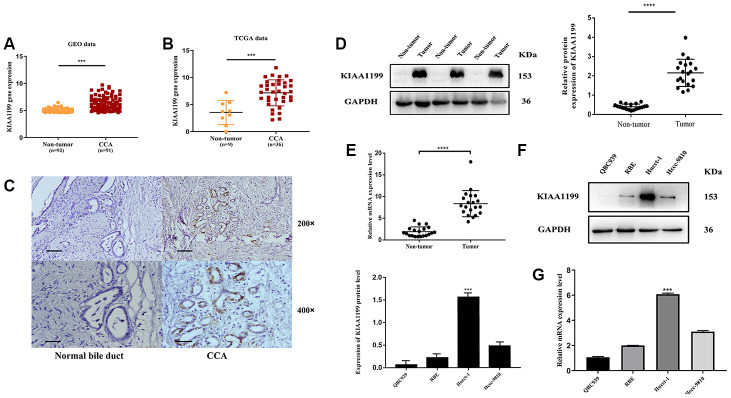
**Bioinformatics prediction.** (**A**) GEO: (GSE76297), Normal: n=92, CCA: n=91, (p<0.001). (**B**) TCGA database, Normal: n=9, CCA: n=36, (p<0.001). Protein and mRNA expression of KIAA1199 in CCA tissues cell lines (**C**) The relative expression levels of KIAA1199 in Pathological sections. (**D**) The relative protein expression levels of KIAA1199 in 20 pairs of CCA and adjacent normal tissues(p<0.001). (**E**) The relative mRNA expression levels of KIAA1199 in 20 pairs of CCA and adjacent normal tissues(p<0.001). (**F**, **G**) The relative protein and mRNA expression of KIAA1199 in four CCA cell lines.>

The TCGA and GEO databases were used to compare KIAA1199 mRNA expression levels in patients with CCA. KIAA1199 expression was significantly higher in CCA patients than in normal subjects (Figure 1A, 1B).

### KIAA1199 expression in human CCA tissues and cell lines

We evaluated KIAA1199 expression in CCA and adjacent tissues from 177 patients by immune-histochemistry. KIAA1199 expression was higher in carcinoma tissues (143/177 (80.8%); Table 1, Figure 1C) than in paracancer tissues (85/177 (48%); Table 1, Figure 1C). In addition, KIAA1199 expression levels were measured in twenty pairs of clinical CCA and paracancer tissues by qPCR and Western blotting. KIAA1199 protein and mRNA levels were higher in CCA tissues than in normal tissues (P<0.001 for KIAA1199; Figure 1D, 1E).

**Table 1:** Differential expression of KIAA1199 in CCA tissues and corresponding paracarcinoma tissues (n=177).

	**KIAA1199 expression**		**P-value**
	**Low (%)**	**High (%)**	
**Carcinoma tissues**	34 (19.2%)	143 (80.8)	<0.001
**Paracarcinoma tissues**	92 (52%)	85 (48%)	

Among the CCA cell lines, HuCCT1, RBE and HCCC9810 cells had the highest and QBC939 cells had the lowest KIAA1199 expression levels (Figure 1F, 1G). Hucct-1 were thus used as the cell model for RNA knockdown and QBC939 were used as the cell model for RNA overexpression.

### Localization of KIAA1199

**Figure 2:**
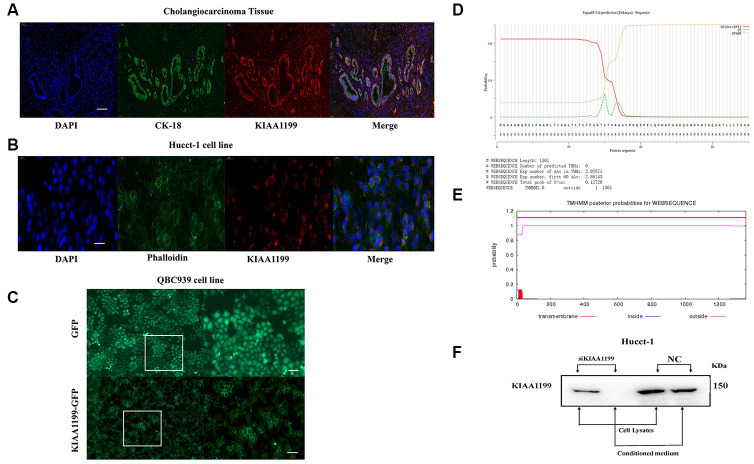
(**A**) Immunofluorescence localization of KIAA1199 and CK-18 in cholangiocarcinoma tissue (red, KIAA1199; green, CK-18; blue, DAPI). (**B**) Immunofluorescence localization of KIAA1199 and Phalloidin in Hucct-1 cell line (red, KIAA1199; green, Phalloidin; blue, DAPI). (**C**) Microscopic determination of KIAA1199 cellular localization using QBC939 cells transfected with green fluorescent protein (GFP), KIAA1199-GFP chimeric cDNAs. (**D**) KIAA1199 had signal peptides. (**E**) KIAA1199 has 602 amino acids, all of which are extracellular, and there is no transmembrane domain (TMD). (**F**) Western blot analysis was performed to detect KIAA1199 in cell lysates and conditioned culturing medium from Hucct-1 and Hucct-1 knockdown KIAA1199 (siKIAA1199).

To unravel the mechanism underlying KIAA1199-mediated cell migration, we determined the subcellular localization of KIAA1199 by employing immunostaining and fluorescent tagging approaches. The Hucct-1 cell line and CCA tissue exhibited high mRNA and protein expression levels of KIAA1199 and were thus used to examine the subcellular localization of endogenous KIAA1199 protein. Immunofluorescence analysis showed that KIAA1199 is localized primarily in the perinuclear space of CCA cells (presumably the ER, including the outer nuclear membrane and ER tubules) and the plasma membrane (Figure 2A, 2B). KIAA1199 was fused with GFP at the C-terminus and transfected into QBC939 cells. We found that KIAA1199 was expressed on the cell membrane. GFP (lack of signal peptide; spread throughout cells) was used as a control (Figure 2C).

The SignalP v5.0 results indicated that KIAA1199 contains signal peptides (Figure 2D). According to TMHMM Server v2.0, KIAA1199 comprises 602 amino acids, all of which are extracellular, and no transmembrane domain (Figure 2E). Thus, the SignalP v5.0 and TMHMM Server v2.0 results also indicated that KIAA1199 is a classically secreted protein.

In addition, large amounts of exogenous KIAA1199 protein were secreted by CCA cells. We detected the KIAA1199 protein in the culture medium of Hucct-1 cells. However, after the expression of KIAA1199 was silenced, the KIAA1199 protein was not detected in the culture medium (Figure 2F).

### KIAA1199 is a secreted molecule and is highly expressed in bile and serum

**Figure 3:**
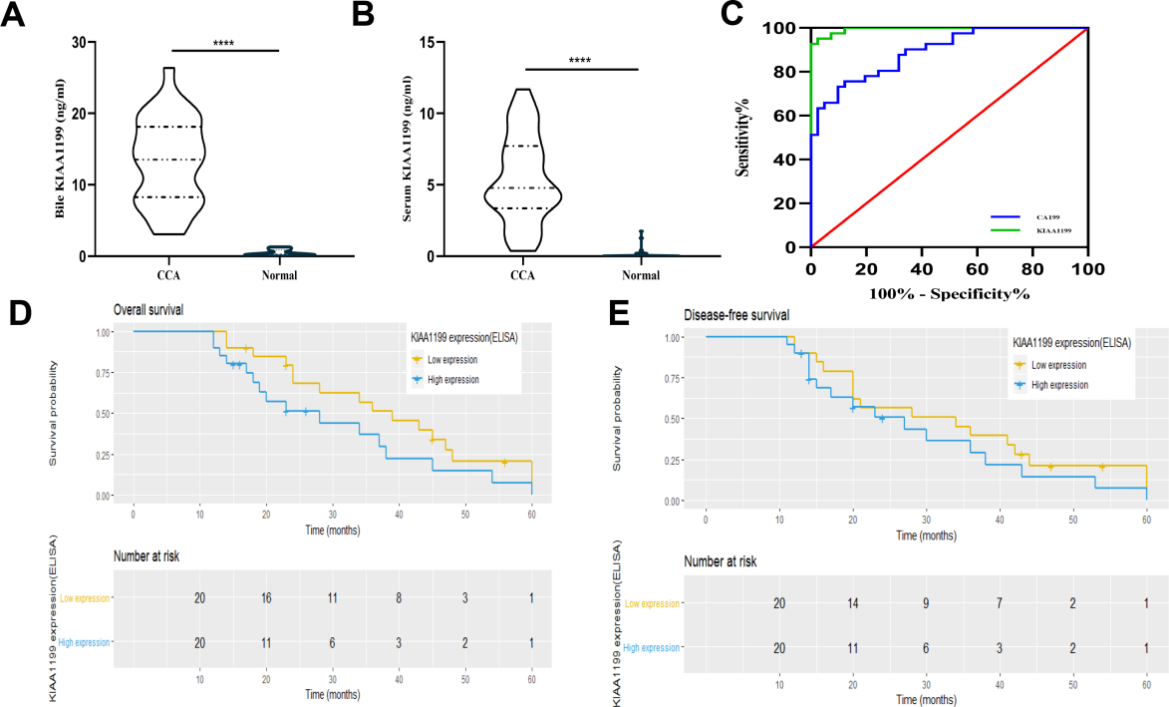
****(**A**) Comparison of bile KIAA1199 levels in CCA patients (n=33) and healthy controls (n=15), p<0.001. (**B**) Comparison of serum KIAA1199 levels in CCA patients (n=41) and healthy controls (n=41), p<0.001. (**C**) ROC curve was used to evaluate serum KIAA1199 and ca199 diagnostic performances. (**D**, **E**) Kaplan-Meier analyses for overall survival (OS) and disease-free survival (DFS) in 41 CCA patients according to serum KIAA1199 levels. The quartile method is used to divide the data into four parts, and the dotted line in the figure is the dividing line of each part.

Then, we used ELISA to detect the expression of KIAA1199 in bile and serum. KIAA1199 levels were high in bile from CCA patients with cholestasis (median: 13.54 ng/ml (range: 3.059-26.39 ng/ml), Figure 3A) and no expression of KIAA1199 was detected in bile of healthy individuals (patients with right hepatic hemangioma or who had undergone cholecystectomy). In addition, we measured KIAA1199 levels in 41 paired serum samples from CCA patients and healthy subjects. As shown in Figure 3B, serum KIAA1199 levels were significantly higher in the CCA patients than in the normal subjects (median: 0.1842 (range: 0-1.75) ng/ml vs. 4.78 (range: 0.365-11.698) ng/ml, P<0.001).

A ROC curve was constructed (Figure 3C). Compared with the traditional diagnostic marker CA199, serum KIAA1199 has obvious diagnostic advantages. The area under the curve (AUC) for serum KIAA1199 is 0.995, and that for CA199 is 0.894.

### Relationship between serum KIAA1199 levels and prognosis

We investigated the relationship between serum KIAA1199 levels and prognosis in patients with CCA. In the serum cohort, we evaluated the preoperative serum KIAA1199 levels in 41 patients with CCA. Most patients had advanced-stage CCA (stage III/IV, n = 29 [70.7%]). We stratified the CCA patients according to their serum expression level of KIAA1199 with a cutoff value of 4.7841 ng/ml, which was the median value in CCA patients. The patients were divided into two groups according to this cutoff value: a high serum KIAA1199 group (n=21) and a low serum KIAA1199 group (n=20). Survival curves were plotted using the Kaplan-Meier method, and survival times were assessed by the log-rank test. The overall survival (OS) times of patients with high serum KIAA1199 levels were significantly shorter than those of patients with low serum KIAA1199 levels (P<0.05, Figure 3D). The median OS times and 36- and 60-month survival rates in the high and low serum KIAA1199 groups were 28 and 39 months, 37.5% and 51.2%, and 6.25% and 18.8%, respectively. Moreover, the disease-free survival (DFS) times of patients with high serum KIAA1199 levels were significantly shorter than those of patients with low serum KIAA1199 levels (P<0.05, Figure 3E). The median DFS times and 12- and 36-month DFS rates in the high and low serum KIAA1199 groups were 27.0 and 34.0 months, 31.5% and 38.5%, and 6.25% and 20.9%, respectively.

Then, we evaluated the relationship between serum KIAA1199 expression and related clinical parameters (Table 2). Serum KIAA1199 levels in CCA patients were markedly related to LN metastasis (P<0.05) and TNM stage (P<0.05). However, the KIAA1199 expression level was not significantly associated with other parameters, for example, age, sex, histological grade, CA19-9 or tumor location.

**Table 2:** Correlations between serum KIAA1199 expression and clinicopathological characteristics in CCA.

**Parameters**	**KIAA1199 expression(ELISA)**		**P-value**
	**High(n=21)**	**Low(n=20)**	
**Age (years)**			
≤60	8	6	0.585
>60	13	14	
**Gender**			
Male	12	13	0.606
Female	9	7	
**Tumor location**			
Intrahepatic	6	5	0.796
Perihilar/ Distal	15	15	
**Histological grade**			
Well	17	16	0.939
Moderate–poor	4	4	
**TNM stage**			
I-II	3	9	0.031
III-IV	18	11	
**Ca19–9(U/ml)**			
≤129	8	9	0.654
>129	13	11	
**Lymph Node Metastasis**			
No	3	11	0.006
Yes	18	9	

### Relationship between KIAA1199 and epithelial-mesenchymal transition (EMT)

**Figure 4:**
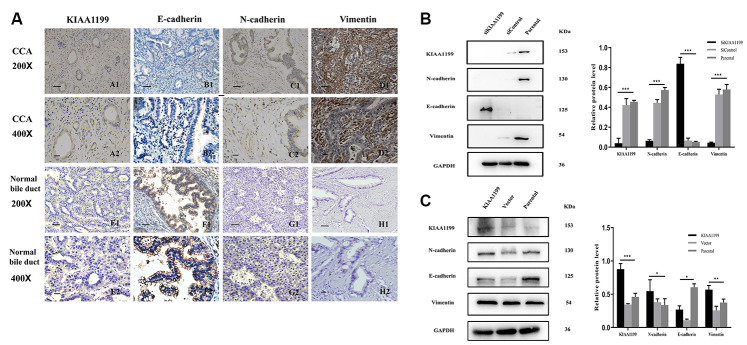
**Immunohistochemical staining for KIAA1199, E-cadherin, N-cadherin and vimentin.** (**A**). A1, A2: Positive KIAA1199 expression in CCA tissue. E1, E2: Negative KIAA1199expression in adjacent tissue. B1, B2: Negative E-cadherin expression in CCA tissue. F1, F2: Positive E-cadherin expression in adjacent tissue. C1, C2: Positive N-cadherin expression in CCA tissue. G1, G2: Positive Vimentin expression in CCA tissue. H1, H2: Negative Vimentin expression in adjacent tissue. (scale bar, 50 μm; magnification: ×200, ×400) (**B**) Western blot analysis of EMT signaling molecules (N-cadherin, E-cadherin and Vimentin) in KIAA1199 silenced Hucct1 cell line. (**C**) Western blot analysis of EMT signaling molecules ((N-cadherin, E-cadherin and Vimentin) in KIAA1199 overexpressed QBC939 cell line. Representative of three independent experiments.

We examined the relationship between KIAA1199 and EMT-related proteins in CCA. The expression of KIAA1199 was higher in carcinoma tissues (Table 1, Figure 4A A1, A2) than in paracancer tissues (Table 1, Figure 4A E1, E2). The expression of E-cadherin was high in 49 of the 177 CCA specimens (27.7%; Table 3, Figure 4A B1, B2). According to Spearman’s rank correlation analysis, KIAA1199 and E-cadherin exhibited a profound negative relationship in the CCA samples (r=-0.371, P<0.001; Table 3). N-cadherin expression was high in 127 of the 177 CCA tissue samples (71.8%; Table 3, Figure 4A C1, C2), and the KIAA1199 and N-cadherin expression levels were significantly positively correlated in CCA specimens according to Spearman’s rank correlation analysis (r=0.311, P<0.001; Table 3). Additionally, vimentin expression was high in 136 of the 177 CCA tissues (76.8%; Table 3, Figure 4A D1, D2), and Spearman’s rank correlation analysis identified a significant positive correlation between the KIAA1199 and vimentin expression levels in CCA tissues (r=0.351, P<0.001; Table 3).

**Table 3:** Correlations of KIAA1199 expression with N-cadherin, E-cadherin and vimentin in CCA.

**Immunoreactivity**	**KIAA1199 expression**		**r-value**	**P-value**
	**Low**	**High**		
**N-cadherin expression**				
Low	20	30	0.311	<0.001
High	14	113		
**E-cadherin expression**				
Low	13	115	-0.371	<0.001
High	21	28		
**Vimentin expression**				
Low	19	22	0.351	<0.001
High	15	121		

The potential effects of KIAA1199 on the expression levels of EMT-related proteins were further investigated in CCA cell lines. When KIAA1199 was knocked down in the HuCCT1 cell line, the protein expression level of E-cadherin was increased, while those of N-cadherin and vimentin were decreased (Figure 4B). In contrast, when KIAA1199 was overexpressed in the QBC939 cell line, the protein expression level of E-cadherin was decreased, while those of N-cadherin and vimentin were increased (Figure 4C).

### Validation in a large sample retrospective cohort

We used a large sample cohort to further validate the effect of KIAA1199 on the prognosis of CCA and to assess the relationship between KIAA1199 expression and clinical parameters in CCA patients. As shown in Table 4, high KIAA1199 expression was associated with the histological grade (P<0.05), LN metastasis (P<0.05), the TNM stage and CA19- level (P<0.05).

**Table 4:** Correlations between KIAA1199 expression and clinicopathological characteristics in CCA.

**Parameters**	**KIAA1199 expression**		**P-value**
	**Negative**	**Positive**	
**Age (years)**			0.446
≤60	13	65	
>60	21	78	
**Gender**			0.786
Male	18	72	
Female	16	71	
**Tumor location**			0.698
Intrahepatic	5	30	
Perihilar/Distal	29	113	
**Histological grade**			0.012
Well	23	60	
Moderate–poor	11	83	
**TNM stage**			0.013
I-II	20	51	
III-IV	14	92	
**Ca19-9(U/ml)**			0.050
≤129	13	81	
>129	21	62	
**Lymph Node Metastasis**			0.012
No	18	43	
Yes	16	100	

**Figure 5:**
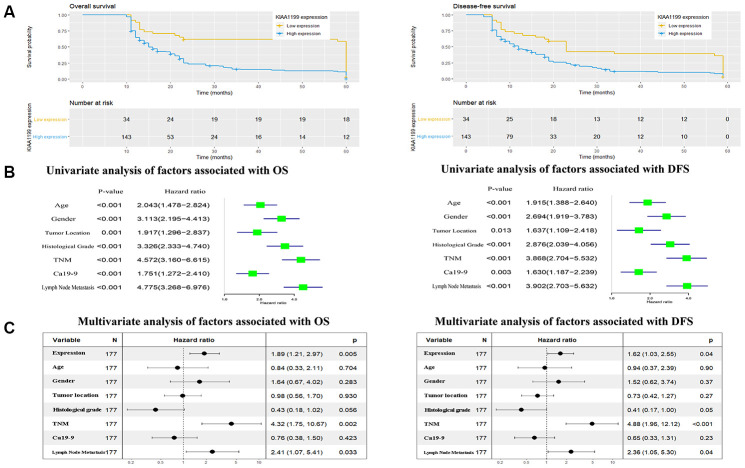
****(**A**) Kaplan-Meier analysis of overall survival (OS) and disease-free survival (DFS) in 177 patients with CCA according to KIAA1199 staining. (**B**, **C**) Univariate and multivariate analyses of factors associated with survival and recurrence.

Survival curves were generated using the Kaplan-Meier method, and differences in the survival times were assessed by the log-rank test (Figure 5A). CCA patients with high KIAA1199 expression had longer DFS and OS times than those with low KIAA1199 expression (P<0.05 12 15 23 56 for both). The median DFS times in the high and low KIAA1199 expression groups were months, respectively. OS respectively.

The univariate analysis results (Figure 5B) showed that age, sex, tumor location, histological grade, TNM stage, LN metastasis and CA19-9 level were associated with both OS and DFS. Multivariate survival analysis with correction (Figure 5C) showed that high KIAA1199 expression, high histological grade, advanced TNM stage and LN metastasis were associated with reduced OS and DFS times. These results indicate that KIAA1199 expression is an independent predictive factor for both DFS (HR=1.62, 95% CI: 1.03-2.55, P<0.05) and OS (HR1.89, 95% CI: 1.21-2.97; P=0.005).

### KIAA1199 promotes CCA cell proliferation, cell migration and invasion

**Figure 6:**
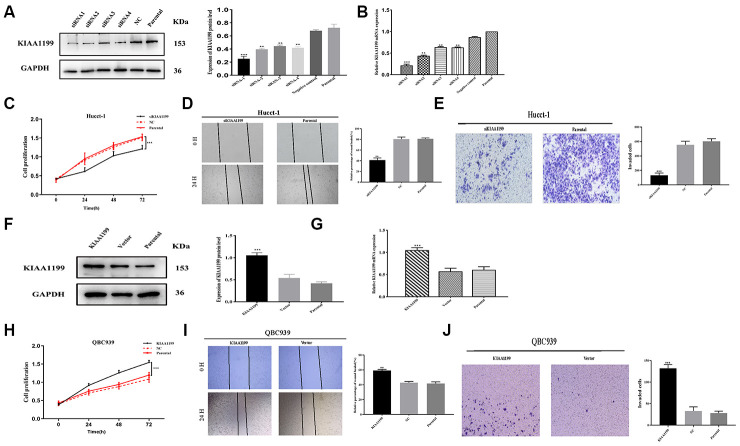
**KIAA1199 regulates proliferation and invasion in CCA cell lines.** (**A**, **B**) The relative protein and mRNA expression of four small interfering RNA in siKIAA1199-transfected cells compared with control and parental cells. (**F**, **G**) Overexpression of KIAA1199 in QBC939 cells with lentivirus infection was verified by western blotting and qPCR. Proliferation of Hucct-1 cells was detected with CCK-8 after silencing KIAA1199 in Hucct-1 cells (**C**) and overexpressing KIAA1199 in QBC939 cells (**H**) in normal medium with 10% FBS. Wound healing assay was applied to evaluate migration of Hucct-1 (**D**) and QBC939 (**I**). 24 h after a scratch in the cell monolayer, the wound size was measured again. Migration of Hucct-1 and QBC939 cells was assessed with transwell assay (**E**, **J**). After KIAA1199 knockdown and overexpression, cells were seeded in the upper transwell chamber and incubated for 24 h, with FBS in the lower chamber. (original magnification: ×200; scale bar, 20 μm). Data, mean ± S.D., and representative of three independent experiments.

To investigate the role of KIAA1199 in vitro, KIAA1199 was knocked down with siRNA. siRNA-1 was selected for further experiments because it yielded the largest reduction in KIAA1199 expression in the transient transfection experiment according to both Western blotting and qPCR (Figure 6A, 6B).

The results of retrospective clinical studies suggest that KIAA1199 is involved in tumor migration and invasion. Therefore, we selected cells with low (QBC939) and high (HuCCT1) KIAA1199 expression for these in vitro experiments. After silencing KIAA1199 with siRNA-1 in Hucct-1 and overexpressing KIAA1199 in QBC939 with lentivirus carrying LV-KIAA1199 (Figure 6F, 6G).

The CCK-8 proliferation assay results showed that KIAA1199 knockdown suppressed Hucct-1 cell proliferation (Figure 6C) and KIAA1199 overexpression promoted QBC939 cell proliferation (Figure 6H). The results of wound healing assays showed that KIAA1199 overexpression enhanced but KIAA1199 knockdown suppressed CCA cell migration (Figure 6D, 6I). Moreover, the results of transwell assays revealed that KIAA1199 upregulation drastically increased but KIAA1199 knockdown dramatically decreased invasiveness (Figure 6E, 6J).

### KIAA1199 promotes CCA growth *in vivo*

**Figure 7:**
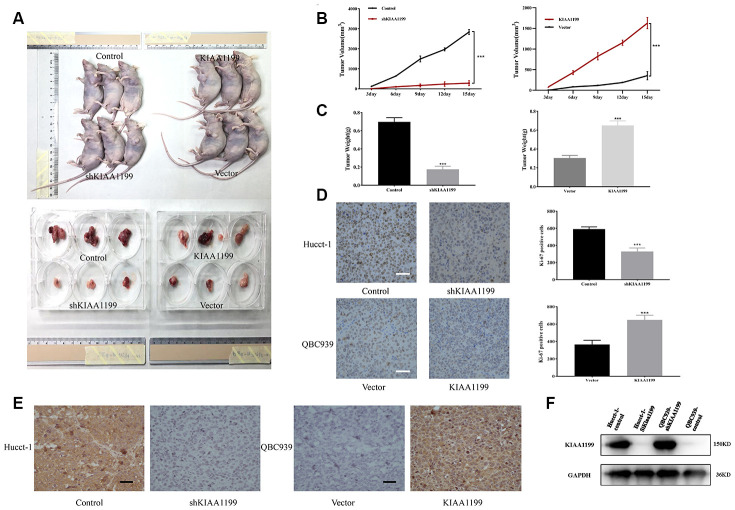
****(**A**) Xenografts were established in nude mice with stable KIAA1199 knockdown or overexpression cell. KIAA1199 knockdown decreased the volume (**B**) and weight (**C**) of xenograft tumors. KIAA1199 overexpression increased the volume and weight of xenograft tumors. Tumor diameter was measured every 3 day. (**D**) Ki67 staining showed that KIAA1199 can improve the proliferation ability of xenograft tumors. (**E**, **F**) Immunohistochemistry and WB detected the expression of KIAA1199 in xenograft tumors. *, ** and *** represented P<0.05, P = 0.01 and 0.001 by Student's t-test, between the indicated groups.

Our studies of clinical data and cell lines indicated that KIAA1199 plays an important role in promoting the proliferation and development of CCA. We then examined the effect of KIAA1199 on tumor growth in a nude mouse subcutaneous xenograft model (Figure 7A). The size and weight of tumors from mice in the shKIAA1199 group were significantly reduced compared to those from mice in the control group (P<0.001). The size and weight of tumors from mice in the KIAA1199 group were significantly increased compared to those vector (P<0.001) (Figure 7B, 7C). KI67 results are consistent with the above results, KIAA1199 overexpression accelerates the proliferation of transplanted tumors (Figure 7D). Immunohistochemistry and WB were then performed to detect KIAA1199 expression in tumor tissues (Figure 7E, 7F).

### KIAA1199 upregulates the TGF-β-PI3K-AKT-mediated EMT signaling pathway

**Figure 8:**
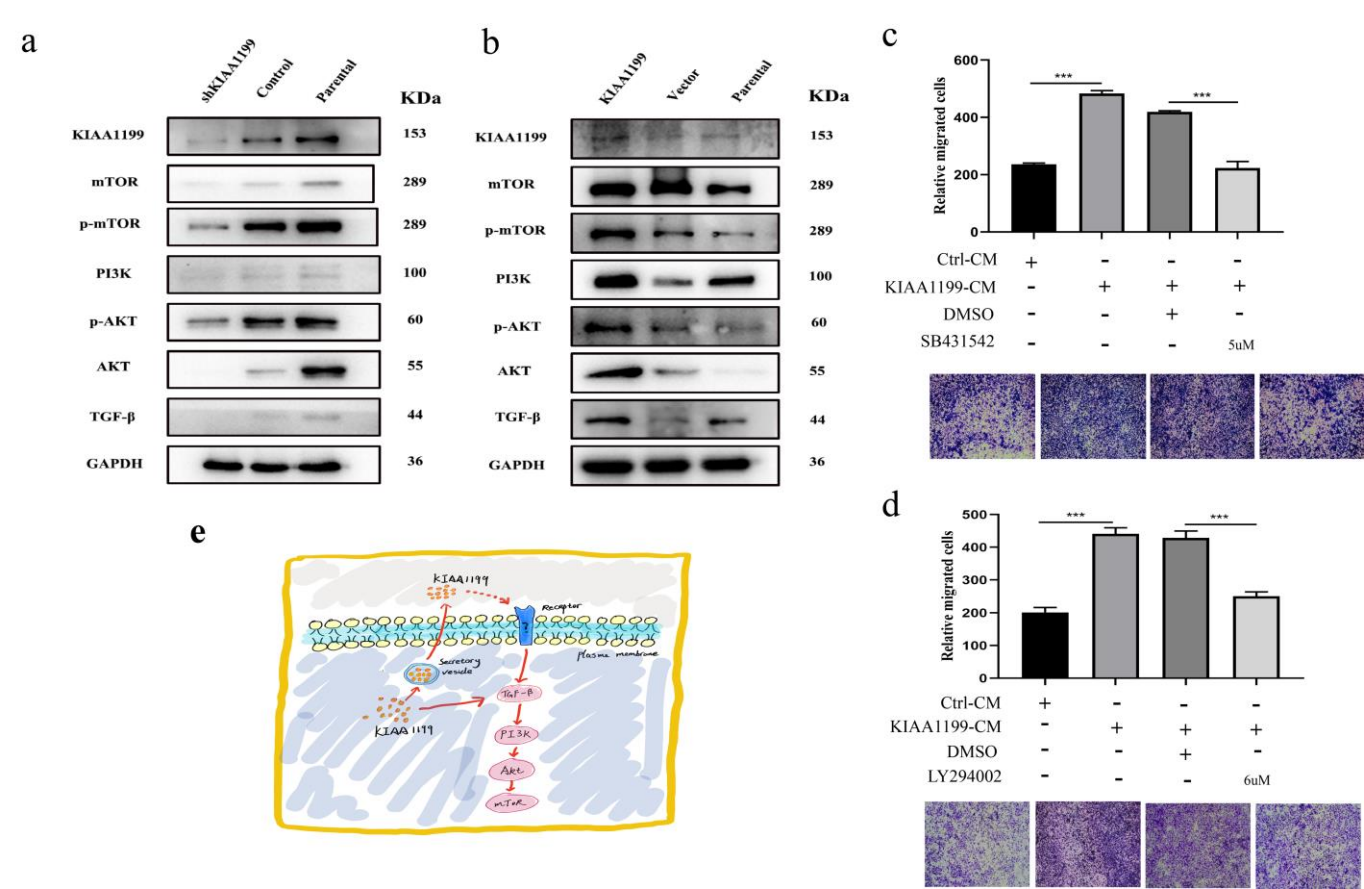
**The expression of KIAA1199 and TGF-β-PI3K-Akt pathway-associated proteins by western blot analyses.** (**A**) Western blot analysis of KIAA1199 and TGF-β-PI3K-Akt pathway-associated proteins in KIAA1199 silenced Hucct1 cell line. (**B**) Western blot analysis of KIAA1199 and TGF-β-PI3K-Akt pathway-associated proteins in KIAA1199 overexpressed QBC939 cell line. (**C**) QBC939 were pretreated with TGF-β inhibitor (SB431542, 5 μM) for 2 h and transwell migration assay was performed in the absence or presence of KIAA1199 conditioned medium (CM). (**D**) QBC939 were pretreated with PI3K inhibitor (LY294002, 6μM) for 2 h and trans-well migration assay was performed in the absence or presence of KIAA1199 conditioned medium (CM). (**E**) KIAA1199-mediated EMT may occur through a non-Smad pathway. At least three independent experiments were preformed, data presented as mean ± SD, *, ** and *** represented P<0.05, P= 0.01 and 0.001 by Student's t-test, between the indicated group.

Alterations in KIAA1199 expression can influence the behaviors, including proliferation and invasion, of CCA tumor cells. The immunostaining and Western blot results indicated that KIAA1199 expression was strongly positively correlated with the expression of EMT-related proteins, such as N-cadherin and vimentin, and was negatively correlated with the expression of E-cadherin. Thus, we measured the expression of proteins in the classical EMT pathway—the SMAD-independent pathway. TGF-β was dramatically downregulated after KIAA1199 silencing, and the TGF-β-regulating proteins PI3k, AKT and mTOR were significantly downregulated in response to KIAA1199 silencing (Figure 8A). In contrast, when KIAA1199 was overexpressed in the QBC939 cell line, the expression levels of TGF-β, PI3k, AKT and mTOR were higher in the OE cells than in the negative control cells (Figure 8B).

To further confirm that KIAA1199 enhances the proliferation and migration of CCA by upregulating the TGF-β-PI3K-AKT-mediated EMT signaling pathway, we treated QBC939 cells with the small molecule TGF-β inhibitor SB431542 and small molecule PI3K inhibitor LY294002. Both SB431542 and LY294002 inhibited KIAA1199-induced migration of cells cultured in conditioned medium (CM) containing KIAA1199 (Figure 8C, 8D).

## DISCUSSION

Jiang [16], Birkenkamp-Demtroder [11] and Matsuzaki [13] studied the role of KIAA1199 in hepatocellular cancer, colorectal cancer and gastric cancer. However, in most studies, the research cohort was too small to allow any meaningful conclusions to be drawn. Moreover, the role of KIAA1199 in CCA is not well understood, and the available research is limited. For the first time, we identified the function and specific mechanism of KIAA1199 in human CCA progression by examining a large patient cohort and performing long-term follow-up evaluations. The present study constitutes a first step toward filling this research gap.

In previous studies [15], KIAA1199 was considered to be a glycosylated protein, localized primarily in the perinuclear space (likely the ER, including the outer nuclear membrane and ER tubules) and plasma membrane. Recently, researchers [19] also found that KIAA1199 has a parallel β-helix repeat (PbH1) domain, which comprises a β-helix repeat containing eight conserved glycine residues, five repeating β-strands, one α-helix and two GG domains. Many G8-containing proteins are often considered integral membrane proteins with signal peptides and/or transmembrane segments [12, 20, 21]. Thus, KIAA1199 may be a secretory factor that participates in extracellular ligand binding and processing. In this study, we provided evidence supporting this hypothesis via predictions with SignalP v5.0 and TMHMM Server v2.0 and detection of KIAA1199 in cell culture supernatant, bile and serum.

Bile is formed by transmembrane molecules expressed in the bile duct, including water channels (e.g., aquaporins), transporters (e.g., SGLT1, a Na+-glucose transporter) and transforming proteins (e.g., SLC4A2, a Cl-/HCO3- exchanger) [22]. These molecules are expressed at the apical and basolateral membranes of cholangiocytes and promote the movement of water, electrolytes and solutes, thus altering bile volume and composition [23]. Cholestasis refers to the retention of normal bile constituents, including toxic bile acids, within the liver. Patients with CCA often develop cholestasis because the tumor blocks the biliary tract. The high level of KIAA1199 detected in bile from CCA patients with cholestasis confirmed that KIAA1199 is a secreted protein. The high level of KIAA1199 detected in preoperative serum from patients with CCA further confirms this conclusion.

According to the findings of our studies, we conclude the following: (1) KIAA1199 is a secreted protein. (2) Serum KIAA1199 levels are higher in CCA patients than in normal individuals. (3) KIAA1199 expression is high in serum and resected tumor tissue. (4) High KIAA1199 levels can be detected in the bile of patients with CCA cholestasis. (5) Cells secrete KIAA1199 into the supernatant, as shown by cell-based experiments, and the amount of secreted KIAA1199 is related to the cellular expression level of KIAA1199 [19]. We speculate that KIAA1199 secreted by CCA tumors is a potential source of KIAA1199 in bile and serum.

Then, we explored the clinical implications of the serum KIAA1199 level in CCA patients prior to curative surgery, because the serum KIAA1199 level may be a marker for predicting poor prognosis in patients with CCA. Analysis of clinical medical records indicated that the diagnostic accuracy of serum KIAA1199 is higher than that of the traditional diagnostic marker CA199. In addition, OS and DFS were shown to differ significantly between the low and high serum KIAA1199 groups.

Patients in the high serum KIAA1199 expression group had worse OS and DFS than those in the low serum KIAA1199 expression group. In a retrospectively reviewed large sample validation cohort, statistical analysis of the clinical information associated with the pathological sections confirmed that KIAA1199 upregulation is associated with poor prognosis. This result was consistent with the serum analysis results and indicates that high KIAA1199 expression is an independent predictor of poor prognosis.

The relationship between the KIAA1199 expression level and the prognosis of patients with CCA prompted us to further investigate the role of KIAA1199 at the cellular level. Our data showed that CCA cell migration and invasion were significantly inhibited after KIAA1199 silencing and confirmed that KIAA1199 overexpression promoted CCA metastasis and invasion. Thus, the expression level of KIAA1199 in CCA cell lines influences their proliferation and migration, indicating that KIAA1199 plays an important role in CCA progression.

Patients with high KIAA1199 expression have poor prognoses, mainly due to the highly metastatic nature of this disease. Cell-based functional experiments again demonstrated the effects of KIAA1199 on cell proliferation and invasion. However, the mechanism by which KIAA1199 promotes tumor metastasis is not clear. During tumor metastasis, shed tumor cells enter the circulatory system by intravasation. Tumor cells in the circulatory system migrate out of the circulatory system by extravasation, enter distant tissues and form tiny metastatic clones; metastatic foci are then formed through proliferation of these cells [24]. Currently, the most studied mechanism is EMT. The role of EMT in CCA metastasis has received increasing attention [24]. EMT has been shown to significantly accelerate the metastasis of epithelial-derived carcinomas such as CCA [25, 26]. During EMT, epithelial tumor cells lose their polarity, their adhesion phenotype is altered to the mesenchymal phenotype, and their invasion and migration capacities are enhanced. Moreover, EMT is an early event in the distant metastasis of tumor cells [27, 28]. EMT can accelerate the growth of tumor cells [29] and promote tumor invasion and metastasis. E-cadherin [30], N-cadherin [31] and vimentin [28] are important protein markers of EMT. Decreased levels of E-cadherin can lead to decreased cell adhesion, thus promoting cell invasion and metastasis, and loss of E-cadherin expression has historically been considered the most prominent feature of EMT [32]. In addition, the expression of vimentin, N-cadherin and other proteins is increased in cells exhibiting the interstitial phenotype [31].

Here, we found that low E-cadherin expression correlates with high KIAA1199 expression in CCA. In contrast, the expression levels of vimentin and N-cadherin were positively correlated with that of KIAA1199 in CCA. Therefore, the expression levels of EMT-related proteins are closely related to high KIAA1199 expression levels in CCA, indicating that KIAA1199 may promote CCA by enhancing EMT.

Numerous signaling pathways mediate EMT. The TGF-β signaling pathway is thought to be the most important signaling pathway for EMT induction during development and in cancer, and other pathological conditions [33]. In some in vitro cultured epithelial cell lines, TGF-β stimulation alone can induce EMT [34]. TGF-β signaling-mediated EMT can be activated by either the classical SMAD-dependent pathway or the SMAD-independent pathway. In the TGF-β signaling-mediated SMAD-independent signaling pathway, PI3K-AKT-mTOR signaling is activated, leading to transcriptional regulation. In addition, activated AKT can trigger EMT by inhibiting the transcriptional regulation of ribonucleoprotein E1 (hnrnpe1) [33]. In this study, results clarify that KIAA1199-mediated EMT may occur via the SMAD-independent pathway (Figure 8E).

Although our research links exogenous KIAA1199 with the proliferation and migration of CCA cells, the specific mechanisms by which KIAA1199 affects CCA cells remain unclear (e.g., whether the effects are autonomous or nonautonomous and whether a KIAA1199 receptor or binding partner is involved).

Although various models have been used to verify the role of KIAA1199 in different cell lines, and the role of KIAA1199 has been examined in xenograft models, the lack of a gene knockout mouse model prohibits further evaluation of the physiological effects of KIAA1199. This inability is another limitation of this study. Moreover, since this study was conducted primarily through retrospective analysis, the results may be biased. Prospective cohorts are needed to further validate our conclusion.

Despite these limitations, our study is the first to demonstrate the potential of KIAA1199 as a novel prognostic biomarker for CCA. In addition, KIAA1199 is a promising new diagnostic molecule and therapeutic target in CCA. Serum KIAA1199 levels are also a potential clinical tool for predicting tumor recurrence and overall prognosis in patients after curative surgery.

## MATERIALS AND METHODS

### Bioinformatics prediction

KIAA1199 expression in CCA was predicted using the cancer-related databases The Cancer Genome Atlas (TCGA) and Gene Expression Omnibus (GEO). The GSE76297 (normal: n=92, CCA: n=91) and TCGA (normal: n=9, CCA: n=36) datasets were downloaded from these databases for differential gene expression analysis in CCA. We used the TCGA database-based tumor data analysis website (UALCAN, http://ualcan.path.uab.edu/) to obtain clinical data analysis results.

### CCA cell lines

The QBC939, HCCC9810, HuCCT1 and RBE cell lines were purchased from Procell Life Science & Technology Corporation (Wuhan, China). CCA cell lines were cultured in RPMI 1640 medium (HyClone, Canada) or DMEM (HyClone, Canada) supplemented with 10% fetal bovine serum (Gibco, NY, USA) and 100 U/ml antibiotic solution (Gibco, NY, USA). The cell incubator was maintained at 37°C with 5% CO2 and 95% humidity.

### Patient enrollment and follow-up

From January 2007 to December 2017, 177 patients undergoing curative resection for pathologically confirmed CCA were enrolled in this study. The patients’ clinical information, including age, sex, tumor location, tumor histological grade, tumor–node–metastasis (TNM) stage, lymph node (LN) metastasis status, and serum carbohydrate antigen 19-9 (CA19-9) level, was recorded. Table 4 lists the detailed clinical information of the 177 CCA patients in this study. Twenty fresh frozen cancer tissues, 41 preoperative serum samples from CCA patients (Table 3), 15 bile samples from healthy individuals (patients with right hepatic hemangioma or who had undergone cholecystectomy) and 33 bile samples from CCA patients were collected between 2010 and 2020. In accordance with the Declaration of Helsinki, the patients provided signed written consent for the collection of bile, serum and tissue samples used in this study. This study was approved by the Medical Ethics Committee of Shandong University and Qilu Hospital of Shandong University.

### Secreted protein prediction

The presence of N-terminal signal peptides and the lack of transmembrane domains (TMDs) are the two major characteristics of secreted proteins. Proteins that meet these two criteria are classified as the computational secretome [35].

We used SignalP v5.0 (http://www.cbs.dtu.dk/services/SignalP/index.php) to identify N-terminal signal peptides and TMHMM v2.0 (http://www.cbs.dtu.dk/services/TMHMM/) to predict transmembrane domains with the default parameters. The KIAA1199 [Homo sapiens] proteome was obtained from the NCBI database (https://www.ncbi.nlm.nih.gov/protein/).

### Measurement of KIAA1199 levels in bile and serum

KIAA1199 levels in human bile and serum samples were measured. Whole blood samples were incubated in a serum separation tube (SST) at 4°C overnight and were then centrifuged at 1000 × g for 15 min. The serum was immediately removed and tested, or the sample was aliquoted and stored at -80°C. Repeated freeze-thaw cycles were avoided. Bile was collected from CCA patients with cholestasis, and the samples were centrifuged for 20 min at 12000 × g and 2-8°C. The levels of KIAA1199 in serum and bile were measured by enzyme-linked immunosorbent assay (ELISA). The KIAA1199 levels were quantified with Human KIAA1199 ELISA kits (SER965Hu, Cloud-Clone, USA and CSB-E13092h, CUSABIO, China).

### Immunohistochemistry

A two-step protocol was used for the immunohistochemical analysis according to the manufacturer’s instructions (SP-9001, ZSGB-BIO; Beijing, China). The pathological sections were dewaxed, subjected to antigen retrieval in a microwave oven, and incubated with the appropriate primary antibodies (diluted 1:100) overnight. Subsequently, the pathological sections were incubated for 30 min with the secondary antibody (SA00004-2; Proteintech, Wuhan, China; diluted 1:400) at room temperature and were stained with diaminobenzidine (DAB Substrate Kit, DA1010; Solarbio, China). Finally, the pathological sections were dehydrated and fixed after counterstaining with hematoxylin. Images were acquired at 200× and 400× magnification with an Eclipse 80i microscope (Nikon, Tokyo, Japan).

The following primary antibodies were used: polyclonal rabbit anti-KIAA1199 (21129-1-AP; Proteintech; Wuhan, China); anti-E-cadherin (20874-1-AP; Proteintech); anti-N-cadherin (22018-1-AP; Proteintech); and anti-vimentin (10366-1-AP; Proteintech), anti-KI67 (27309-1-AP; Proteintech).

Semiquantitative methods were used to quantify the immunohistochemical results, namely, the staining intensity (absent, weak, moderate, strong=0, 1, 2, 3) and percentage of stained cells (<10%, 10%–50%, <50%=1, 2, 3). A total score of <3 for the sum of scores two parameters was considered to indicate low expression. A total score ≥3 high. All results were scored independently by experienced pathologists.

### Immunofluorescence

Frozen sections (4-μm thick) of liver tissue were prepared for immunofluorescence staining. In brief, sections were stained by overnight incubation with primary antibodies, such as anti-KIAA1199 (Proteintech, 1:200), anti-CK18 (Abcam, 1:200), and anti-phalloidin (Abcam, 1:200). Alexa 488-conjugated goat anti-rabbit (A11008, Thermo Fisher; 1:1000) or Alexa 568-conjugated goat anti-mouse (A-21043, Invitrogen; 1:1000) secondary antibodies were used. After three washes with PBST, the slides were incubated with DAPI (Beyotime, 1:2000) for 5 min. In addition, LC3 lentivirus was transfected into CCA cells according to the manufacturer’s recommendations. Images were acquired with a fluorescence microscope (LSM 780, Carl Zeiss).

### Western blot analysis

When cells reached ~80% confluence, the complete medium was removed, and the cells were washed with PBS and cultured in serum-free medium for 8 h. The medium was then collected and supplemented with 1 mM PMSF and 1× EDTA-free complete Protease Inhibitor. The mixture was centrifuged at 100 × g for 5 min to remove suspended cells and was then centrifuged at 3000 × g with an Amicon Ultra centricons (10 kDa NMWL) to concentrate the clarified medium.

Proteins were extracted from CCA cell lines or tissues with radioimmunoprecipitation assay (RIPA) buffer (89900, Thermo Scientific, California, USA) containing protease and phosphatase inhibitors (36978, Thermo Scientific, California, USA). Protein concentrations were measured according to the kit manufacturer's instructions (Beyotime BCA Protein Assay Kit, P0009, China). Then, proteins were separated by 10% SDS-PAGE and transferred to polyvinylidene fluoride (PVDF) membranes (Millipore, NY, USA). Membranes were blocked with 5% BSA blocking buffer (SW3015, Solarbio, Beijing, China) and incubated with primary antibodies (diluted 1:1000) overnight at 4°C. Membranes were then incubated for 1 h with the secondary antibody (diluted 1:5000), and the protein bands were visualized with a Western Fluorescence Kit (BeyoECL Plus, Beyotime, P0018S, China) and a Chemiluminescence Imaging System (T-4600, Tanon, Shanghai, China). Band densities were quantified with ImageJ, and the target protein levels were normalized to those of GAPDH.

### Quantitative RT-PCR (qPCR)

Total RNA was extracted from cell lines and tissues using a FastPure Cell/Tissue Total RNA Isolation Mini Kit (RC101, Vazyme, Nanjing, China) according to the manufacturer’s protocol. The RNA concentration was determined, and total RNA was reverse transcribed to cDNA (P113-01, Vazyme, Nanjing, China). qPCR was conducted using SYBR Green reagent (FP303, TIANGEN, Beijing, China) in a sequence detection system (ABI 7900HT, Applied Biosystems, MA, USA). The 2^-ΔΔCt^ method was used to analyze the experimental results. All primer sequence information is shown in Table 5.

**Table 5:** Related sequences of KIAA1199.

	**Sequence**
**KIAA1199 F**	5′ -AGGCGTGACACTGTCTCGGCTACAG-3′
**KIAA1199 P**	5′ -CCACTCCACGTCTTGAACCCAC-3′
**GAPDH F**	5′ -TTGGTATCGTGGAAGGACTCA-3′
**GAPDH R**	5′ -TGTCATCATATTTGGCAGGTT-3′
**siRNA-1**	5′-GCAATCGTCCCATTGATATAC-3′
**siRNA-2**	5′-GCTGCAGGATCTGAGGAAACT -3′
**siRNA-3**	5′-GGTTATGACCCACCCACATAC -3′
**siRNA-4**	5′-GGGATAAGACATCTGTGTTCC-3′
**NC(negative control)**	5′-TTCTCCGAACGTGTCACGT-3′

### KIAA1199 silencing and OE

For silencing, a small interfering RNA (siRNA) against KIAA1199 was designed. siRNA was transfected into CCA cell lines with Xfect™ RNA Transfection Reagent (631450, Takara, USA) according to the manufacturer’s instructions. The target siRNA sequences are listed in Table 5. The lentiviral vectors overexpressing KIAA1199 (LV-KIAA1199) and the corresponding knockdown vectors (LV-shKIAA1199) were synthesized by Jinan Boshang Corporation (Jinan, China). Empty vectors were used as the corresponding controls.

### In vitro

We used CCK-8 (Selleck, USA) assays to measure the proliferation of related carcinoma cell lines. The absorbance at 490 nm was measured at 0 h, 24 h, 48 h, and 72 h. Wound healing assays were used to evaluate cell migration. The migrated cells at the edge of the wound were imaged at 0 and 24 h after wounding, and migration was assessed as follows: (average wound area at 24 h - average wound area at 0 h)/average wound area at 0 h. Transwell assays were used to evaluate cell invasion. Transwell inserts (Corning, USA) with an 8-μm pore size were placed in 24-well plates. After incubation for 24 h at 37°C in a 5% CO2 atmosphere, the noninvasive cells remained on the upper surface of the filter, and the highly invasive cells that invaded through the filter were fixed with methanol and stained with crystal violet. Images were acquired at 200× magnification with an Eclipse TS100 microscope (Nikon, Tokyo, Japan).

### In vivo

Twelve nude mice were randomly divided into 4 groups of 3 mice each. Hucct1-control, Hucct1-siKIAA1199, QBC939-control, and QBC939-shKIAA1199 CCA cells were suspended separately in sterile PBS, and these cell suspensions were injected into the left axilla of the nude mice. Each nude mouse was injected with 200 μl of suspension containing approximately 5 × 10^6^ cells. The length (L) and width (W) of the tumors were measured using a Vernier caliper every three days, and the tumor volume (TV) was calculated according to the following formula: TV = (L × W^2^) /2. The nude mice were sacrificed 15 days later, and the tumor tissues were completely resected and weighed. KIAA1199 expression in tumor tissues was detected via Western blotting.

### Statistical analysis

Statistical analysis was performed using SPSS 16.0 (Chicago, USA) and GraphPad Prism v. 6.01 (San Diego, CA, USA). The values are expressed as the mean ± standard error of the mean. Receiver operating characteristic (ROC) curve analysis was used to evaluate the sensitivity and specificity of serum KIAA1199 for diagnosing CCA. An unpaired t-test was used to compare quantitative variables. Pearson’s χ^2^ test or Fisher’s exact test was used to compare qualitative variables. The patient survival curves were plotted using the Kaplan-Meier method, and the log-rank test was used to identify significant differences among groups. Multivariate analysis was performed with a Cox regression model. P<0.05 was considered to indicate a statistically significant difference.
